# Glucocorticoid-Induced Cardiomyopathy Caused by Uncontrollable Asthma

**DOI:** 10.7759/cureus.43780

**Published:** 2023-08-20

**Authors:** Hiroki Uehara, Masaki Okuyama, Yutaro Oe, Takaki Yoshimura, Takahiro Gunji

**Affiliations:** 1 Cardiovascular Medicine, Kin-ikyo Chuo Hospital, Sapporo, JPN

**Keywords:** asthma, reversible cardiomyopathy, steroid use, dilated cardio, heart failure

## Abstract

Hypercortisolism is a risk factor for adverse cardiovascular and cerebrovascular outcomes, including hypertension, hyperglycemia, and dyslipidemia. It has been suggested that cardiovascular risk increases with increasing steroid use in patients taking oral steroids as immunosuppressive drugs. Cardiomyopathy is often reported to occur concomitantly in patients with Cushing's syndrome. Reports of cases of long-term high-dose glucocorticoid ingestion and concomitant cardiomyopathy are rare. We report a case of cardiomyopathy in a 63-year-old Japanese man. He had refractory bronchial asthma and had been on prednisolone ≥15 mg/day equivalent for >20 years. Echocardiography showed severe left ventricular dilatation, left ventricular systolic dysfunction, and mitral regurgitation. Since other secondary cardiomyopathies were excluded, a diagnosis of glucocorticoid cardiomyopathy was made, cardioprotective drugs were introduced, and the steroid dose was reduced during hospitalization. Four months after the patient's discharge, echocardiography showed normalization of left ventricular systolic function.

## Introduction

Oral glucocorticoids provide effective treatment not only for immunocompromised patients but also for various diseases. Steroids should be administered early in the course of an attack in asthmatic patients, after beta-stimulants. Steroids can also be used as maintenance therapy for refractory asthma. However, prolonged oral glucocorticoid treatment can increase the risk of adverse events, including cardiovascular disease [1], and the cardiovascular risk from oral glucocorticoid intake has been reported to be dose-dependent [2,3]. We report a case of cardiomyopathy triggered by exogenous steroid overdose in a background of poorly controlled asthma.

## Case presentation

**Figure 1:**
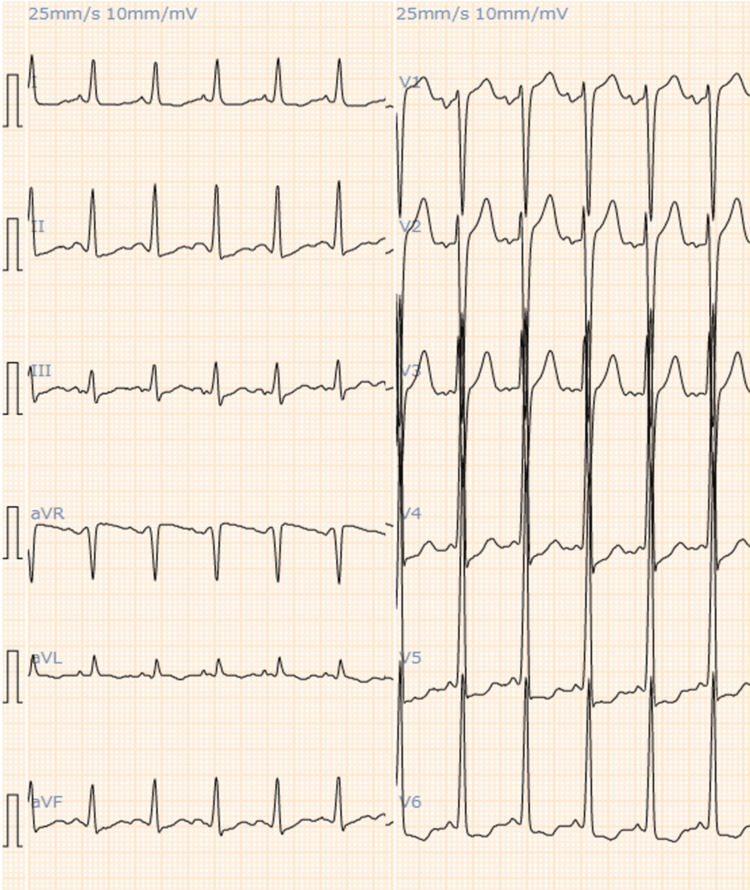
Electrocardiography showing sinus tachycardia and left atrial load findings

A 63-year-old Japanese male presented to our emergency department with respiratory distress symptoms. He is 184 cm tall and weighs 66 kg. There was no family history of heart disease. His medical history included aspirin-induced asthma and polyarticular gout attacks. His asthma was poorly controlled, and he had experienced multiple asthma attacks per month, each time receiving 4 mg of betamethasone intravenously. At the time of this visit, the patient was thought to be having a usual asthma attack, but his respiratory distress symptoms did not improve even after the administration of betamethasone. His blood pressure was 129/96 mmHg, and his heart rate was 136 beats/minute. Chest X-rays showed an enlarged heart and pulmonary congestion. The cardiothoracic ratio (CTR) was 55.4%. Two years ago, chest X-rays were normal, and CTR was 45.5%. Electrocardiography showed sinus tachycardia and left atrial load findings (Figure 1).

This respiratory distress attack was thought to be heart failure rather than asthma. A loop diuretic was thus administered, and the patient was admitted to our hospital for close examination.

**Figure 2:**
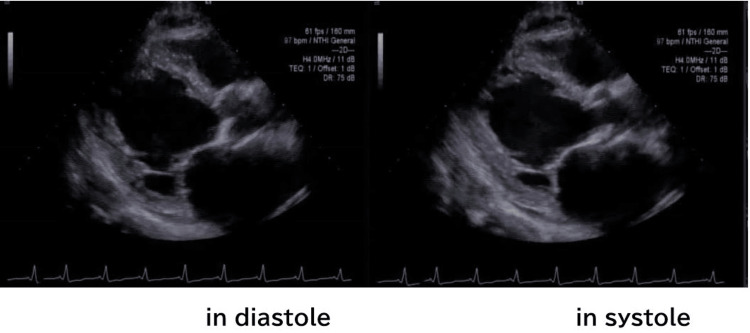
Echocardiography showing left ventricular systolic dysfunction The LVEF was 20%, and the LAD was 58 mm (normal: 30-35 mm). The LVDd was 69 mm (normal: 41-47 mm), and the LVDs were 63 mm (normal: 18-32 mm). The myocardial hypertrophy was mild and the IVSTd was 11 mm (normal: 7-9 mm). The LVPWd was 12 mm (normal: 6-10 mm). Tethered mitral valve and severe mitral regurgitation were noted. LVEF: Left ventricle ejection fraction, LAD: Left atrial dimension, LVDd: Left ventricular internal dimension in diastole, LVDs: Left ventricular internal dimension in systole, IVSTd: Intraventricular septum thickness at end-diastole, LVPWd: Left ventricular posterior wall thickness at end-diastole

He was prescribed prednisolone 15 mg/day for refractory asthma. The amount of steroids used per month is not certain, but he had been using steroids for over 20 years. He also had asthma attacks over the course of a month and received 4 mg to 6 mg of betamethasone IV per attack. Extensive interviews and inquiries to the family physician revealed no previous indication of heart failure, and repeated chest X-rays had all shown normal findings. The patient's usual blood pressure, including during asthma attacks, was approximately 130/90 mmHg. On physical examination, there was no edema in either lower leg and no obvious arthralgia, peripheral neuropathy, or skin rash. Echocardiography showed left ventricular systolic dysfunction. The left ventricle ejection fraction (LVEF) was 20%, and the left atrial dimension (LAD) was 58 mm. The left ventricular internal dimension in diastole (LVDd) was 69 mm, and the left ventricular internal dimension in systole (LVDs) was 63 mm. The myocardial hypertrophy was mild, and the intraventricular septum thickness at end-diastole (IVSTd) was 11 mm. The left ventricular posterior wall thickness (LVPWd) was 12 mm. A tethered mitral valve and severe mitral regurgitation were noted (Figure 2).

Blood samples showed an N-terminal prohormone of brain natriuretic peptide (NTproBNP) level at 1381 pg/mL and troponin T at 0.165 ng/mL; the adrenocorticotropic hormone (ACTH) was 87.4 pg/mL, and cortisol was 5.8 µg/dL. The patient's HbA1c was normal, and various autoantibodies including anti-neutrophil cytoplasmic antibody (ANCA) were also negative. Eosinophils were hyperplastic, accounting for 15.1% of leukocytes, and the total eosinophil count was 1630/µL. Hypereosinophilia was identified about a year ago. All other autoantibodies suggestive of collagen disease were negative. A bone marrow biopsy showed mild eosinophilia but no other abnormal findings.

**Figure 3:**
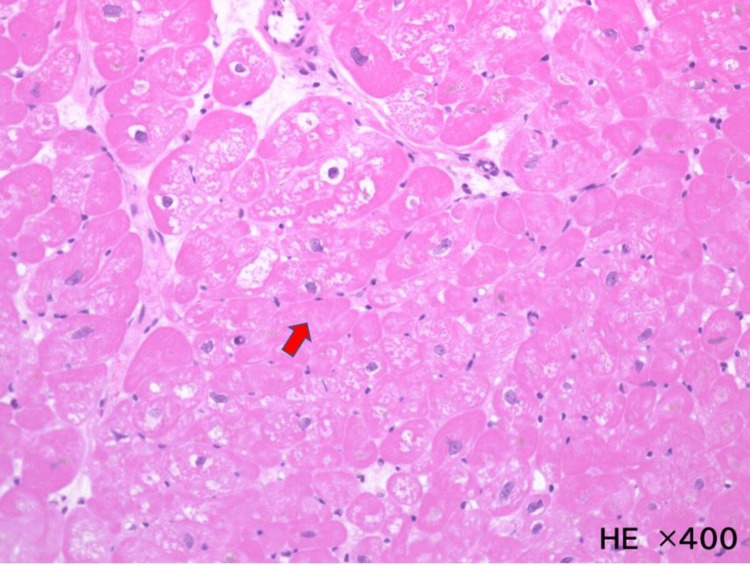
Myocardial biopsy The pathology specimen showed no myocardial convoluted arrangement or fibrosis, but it showed thickening of myocardial cells (red arrow), hematoxylin & eosin staining, ×400.

A cardiac MRI was not performed, but a contrast-enhanced CT of the heart showed no obvious left ventricular delayed contrast. Coronary angiography was performed, and no stenosis was found in the coronary arteries. A myocardial biopsy was performed from the left ventricular posterior wall, and the pathology showed no inflammatory cell infiltrate, no myocardial disarray, and no fibrosis. However, hypertrophy of cardiomyocytes was observed (Figure 3).

Medication was administered to treat heart failure. In parallel with furosemide treatment, bisoprolol combined with sacubitril valsartan, spironolactone, and dapagliflozin were initiated. Because sinus tachycardia was still present after the introduction of a beta-blocker, ivabradine was also started. A bone marrow biopsy was performed for eosinophilia, revealing no significant increase in blasts in the bone marrow. The patient was discharged home with a plan to participate in our outpatient cardiac rehabilitation program. He also started attending our respiratory outpatient clinic for his asthma.

After discharge from the hospital, the patient's treatment course for heart failure went well, and he showed no symptoms of respiratory distress. The cardiac enlargement on chest X-ray also improved, and the loop diuretic was discontinued. As part of the patient's asthma treatment, inhalers were adjusted, and prednisolone was tapered from 15 mg/day to 5 mg/day. The number of outpatient infusions of betamethasone also decreased. Four months after the patient's discharge, echocardiography showed that his LVEF had almost normalized to 53% and the mitral regurgitation had improved to mild status; the LAD was 43 mm, the LVDd was 58 mm, and the LVDs were 42 mm. The IVSTd was 11 mm, and the LVPWd was 11 mm. Six months later, blood samples revealed that NTproBNP had improved to 145 pg/mL.

## Discussion

Our patient's reversible cardiomyopathy associated with the therapeutic intake of steroids was a case of glucocorticoid-induced cardiomyopathy. Steroid cardiomyopathy due to an overdose of anabolic steroids used for muscle gain has been reported [4], but the main ingredient of anabolic steroids is testosterone, which should be considered different from prednisolone and betamethasone, which were overdosed in our patient's case. His reversible cardiomyopathy due to glucocorticoid overdose is thus extremely rare.

As mentioned earlier, hypercortisolism is a risk factor for cardiovascular and cerebrovascular outcomes, including hypertension, hyperglycemia, and dyslipidemia, and the cardiovascular risk from glucocorticoid use has been shown to be dose-dependent [2,3]. One mechanism that may underlie the cardiac effects of steroids is their involvement in the renin-aldosterone system, which can induce myocardial remodeling. The effects of glucocorticoids on cardiomyocytes were studied in a mouse model by Oakley et al. [5], showing that glucocorticoid receptor (GR) signaling in cardiomyocytes is critical for the normal development and function of the heart. In contrast, mineralocorticoid receptor signaling in cardiomyocytes participates in the development and progression of cardiac disease.

Our search of the relevant literature identified no reports of dilated cardiomyopathy due to glucocorticoid overdose, but there are a few reports of cardiomyopathy induced by Cushing's syndrome. Sheikh et al. described a case of cardiomyopathy secondary to Cushing's disease due to a pituitary adenoma [6]. Diuretic therapy and endocrinology have improved the patient’s left ventricular systolic dysfunction. The case report by Frustaci et al. discusses the pathology of cardiomyopathy associated with Cushing's syndrome [7]; they noted that the patient's cardiomyocyte hypertrophy, myofibrillolysis, and myocardial fibrosis were improved at the one-year follow-up after adrenalectomy. Miao et al. analyzed a total of 19 cases of Cushing's syndrome with cardiomyopathy: dilated cardiomyopathy (n=15, 78.94%) and hypertrophic cardiomyopathy (n=4, 21.05%) [8].

Our patient had no history of hypertension or diabetes mellitus. No atherosclerotic changes in the vessels, including the coronary arteries, were identified. Since his blood pressure at previous emergency room visits was also in the normal range, myocardial damage due to hypertension was considered unlikely. Based on our patient's history, the physical examination and examination of the heart and other secondary cardiomyopathies were negative. Using these results together with the findings of the myocardial biopsy (thickening of myocardial cells), we were able to obtain the diagnosis of glucocorticoid-induced cardiomyopathy. Improvement in the patient's cardiac function with renin-angiotensin-aldosterone system (RAAS) inhibitors and steroid reduction was also considered suggestive of glucocorticoid-induced cardiomyopathy.

However, there are a few concerns to consider. The first is that our patient's eosinophils were hyperplastic. The results of peripheral blood draws and the bone marrow biopsy were negative for myeloproliferative disease. The myocardial biopsy revealed no eosinophil infiltration of the myocardium. Since eosinophils have been increasing for a year, active eosinophilic myocarditis was ruled out, but there may have been an eosinophilic influence on cardiomyopathy. A second concern is that the possibility of accompanying eosinophilic granulomatosis with polyangiitis (EGPA) cannot be ruled out. The patient's physical examination revealed no skin rash, and blood samples were negative for myeloperoxidase (MPO)-ANCA. The MPO-ANCA has been reported to be 50% negative in cases of EGPA in Japan [9], and our patient was also taking prednisolone 15 mg/day when the physical examination was performed. Considering these factors, the patient should be reevaluated after discontinuing steroids in order to rule out EPGA.

A third concern is the possible complication of adrenal insufficiency. Dilated cardiomyopathy associated with adrenal insufficiency can improve cardiac function with cortisol replacement [10]. In our patient's case, blood samples taken at the time of presentation showed relatively high serum ACTH and slightly low serum cortisol. It is possible that the patient may have been in a state of recurrent temporary adrenal insufficiency due to frequent loading of steroids. A review of inhaled medications and the introduction of molecularly targeted agents should have been considered at an early stage to treat the patient's bronchial asthma and reduce the steroid dose.

## Conclusions

Hypercortisolism is a risk factor for cardiovascular and cerebrovascular outcomes, including hypertension, hyperglycemia, and dyslipidemia, and the cardiovascular risk from glucocorticoid use has been shown to be dose-dependent. In addition, glucocorticoid overdose can cause reversible cardiomyopathy by itself. When treating patients on high-volume, long-term steroids, efforts should be made to reduce the dose of steroids as soon as possible. Hypercortisolism should also be considered when a patient with cardiomyopathy of unknown cause is encountered.
